# Surgicel on the post-operative CT: an old trap for radiologists

**DOI:** 10.1259/bjrcr.20190041

**Published:** 2019-11-15

**Authors:** Flavius Parvulescu, Gaurav Sundar, Milind Shortri

**Affiliations:** 1 Radiology Department, Aintree University Hospital, Liverpool, UK; 2 General Surgery Department, Aintree University Hospital, Liverpool, UK

## Abstract

Percutaneous drainage of post-operative collections following abdominopelvic surgery has become standard practice and is a routine procedure in many interventional radiology (IR) departments. Such collections are commonly diagnosed on CT studies where the presence of Surgicel ^®^ can mimic an abscess and lead to unnecessary procedures. We present a case where a duodenal perforation was masked by post-operative Surgicel in the gallbladder fossa, which in turn was mistaken for an infected biloma and referred for percutaneous interventional radiology drainage. Careful imaging review, correlation with operative notes and good diagnostic radiological technique led to a correct diagnosis and avoided unnecessary intervention.

## Introduction

Image-guided percutaneous drainage of post-operative collections following abdominopelvic surgery has become standard practice and is a routine procedure in many interventional radiology departments. Such collections are commonly diagnosed on CT studies, where the presence of Surgicel^®^ can mimic an abscess and lead to unnecessary invasive procedures.^1^

Surgicel^®^ (oxidized regenerated cellulose) is an absorbable haemostatic material that has been used in surgery for over 50 years,^2^ most commonly for generalised oozing.

## Case report

**Figure 1.**
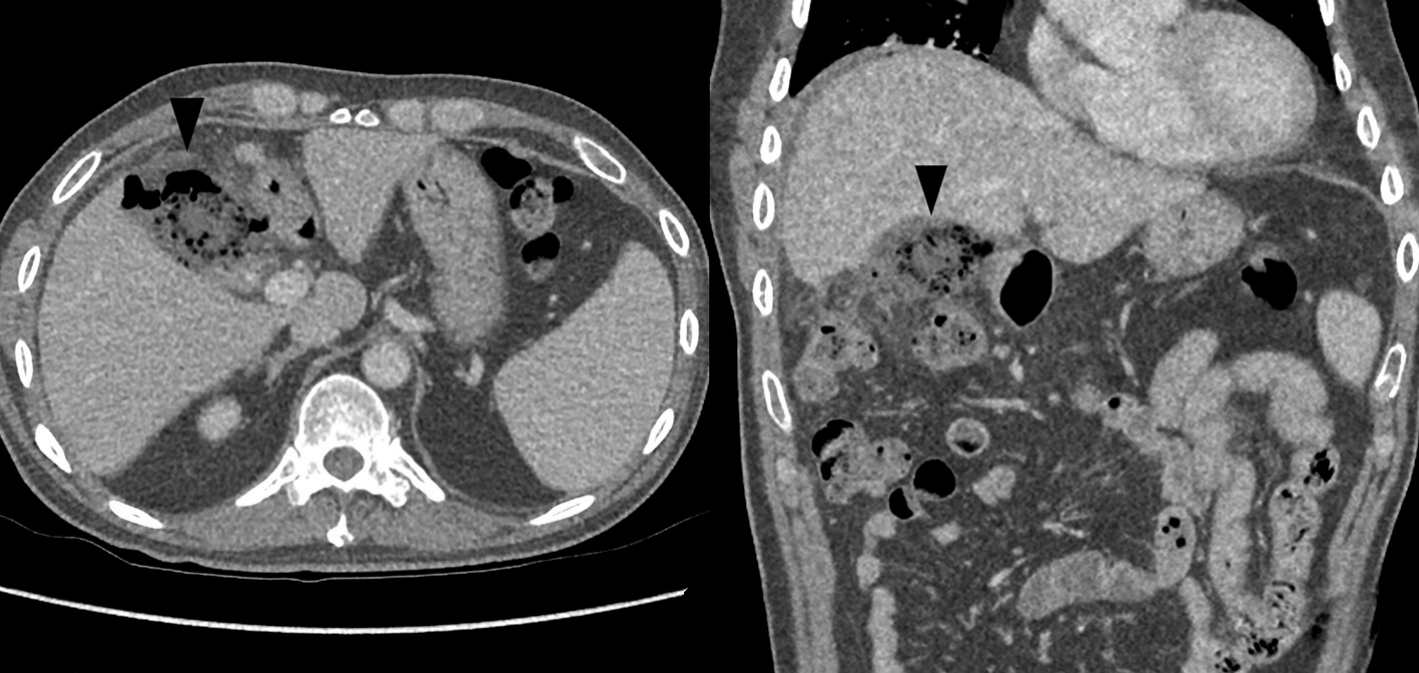
Axial (left) and coronal (right) images from the initial CT study, showing a pseudoabscess in the gallbladder bed (black arrowhead), later proved to be Surgicel^®^. Note the somewhat linear distribution of tightly packed gas bubbles, the absence of enhancing walls and the absence of well-defined air–fluid levels, all of which have been described as favouring haemostatic material over abscess.

A 62-year-old male had been diagnosed on ultrasound with cholelithiasis complicated by chronic cholecystitis when he had presented with epigastric pain. An interval elective cholecystectomy was planned as a day case procedure. This was initially undertaken laparoscopically but due to adhesions from chronic inflammation, resulted in a subtotal cholecystectomy and the surgical bed was packed with Surgicel. The procedure was tolerated well, and the patient was discharged home the same day, with a follow-up appointment planned for 6 weeks later. However, 2 days later, the patient was readmitted with right upper quadrant pain, rigors and raised inflammatory markers: CRP 286 mg l^−1^ (normal range 0–10 mg l^−1^) and WCC 12.4 × 10^9^/L (normal range 4–11 × 10^9^/L). An infected biloma was clinically suspected and a contrast-enhanced abdominopelvic CT was requested. This first CT-study showed the Surgicel as a mixture of fluid attenuation and air locules in the gallbladder fossa (Figure 1). This was described by the reporting radiologist as a “complex localised collection in the gallbladder bed” and misinterpreted as an infected biloma. This was based on limited clinical information, that did not include any intraoperative details.

**Figure 2.**
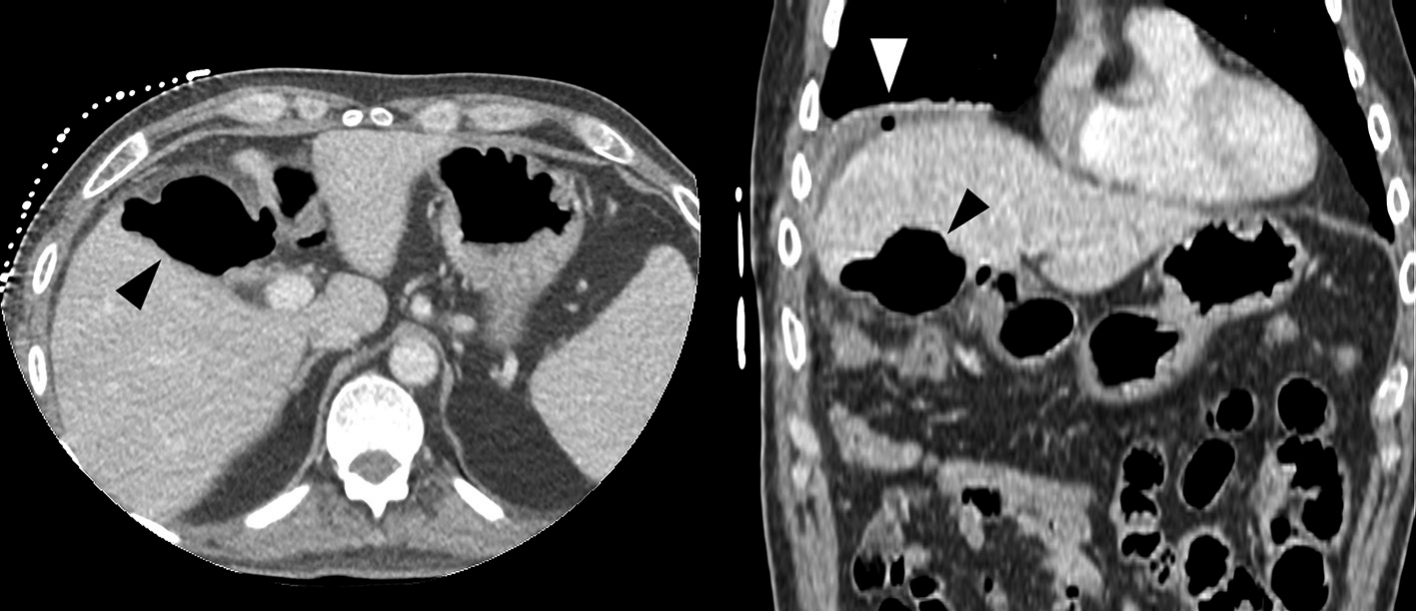
Axial (left) and coronal (right) images from the second CT-study, on the seventh post-operative day, showing complete resorption of the haemostatic material (black arrowhead). Note a small locule of free intraperitoneal gas (white arrowhead) on the coronal image.

Over the following days, after treatment with intravenous antibiotics, the patient showed signs of clinical improvement. However, because of persistently raised inflammatory markers and tachycardia, the surgical team requested percutaneous drain insertion of the presumed biloma. Based on the previous report, the case was vetted by an interventional radiologist and the patient presented for percutaneous drainage, 5 days after the initial CT. As part of the planning for percutaneous drainage, a non-contrast abdominal CT was performed, with the patient supine; this showed a large pocket of gas in the gallbladder fossa, replacing the previously misdiagnosed biloma, as well as subtle pneumoperitoneum (Figure 2).

**Figure 3.**
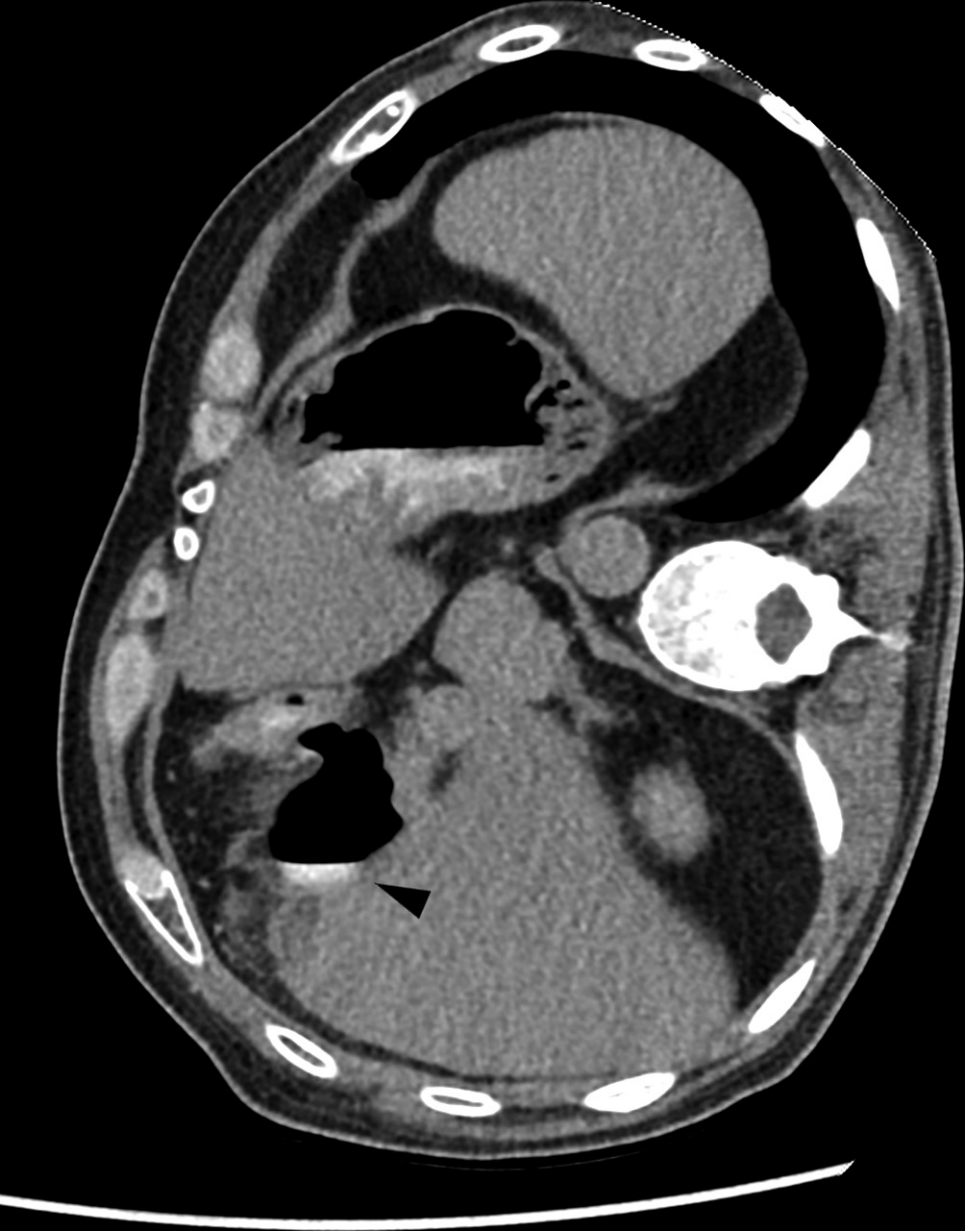
Axial image from the final CT-study, performed in right lateral decubitus, following oral contrast administration. Note extravasation of oral contrast from the gastrointestinal tract into the gallbladder fossa (black arrowhead).

These appearances raised suspicion that the collection previously thought to represent a biloma, in fact represented Surgicel, which had now been absorbed. Moreover, the presence of a large air pocket in the gallbladder fossa and pneumoperitoneum at 1 week post-surgery indicated hollow viscus perforation. In order to confirm this, diluted contrast material was administered orally, and a repeat CT study of the abdomen was performed, in prone position—this showed no extravasation; therefore, it was repeated in right lateral decubitus position. This clearly showed oral contrast pooling within the gallbladder fossa, confirming the suspicion of a duodenal perforation (Figure 3).

No further imaging or intervention was performed. The findings were discussed with the surgical team and conservative management was agreed, as the perforation was thought to be small. The patient was continued on antibiotic treatment for another 5 days and made a good recovery without further incidents.

## Discussion

The imaging appearances of Surgicel^®^ and its resemblance to a postoperative abscess have been described in the literature more than two decades ago, both on CT^3^ and on ultrasound.^4^ Previous case reports presenting such similarities on postoperative CT-studies^1,5^ demonstrated how knowledge of the use of Surgicel^®^ can explain the CT appearances, can decrease the rate of misdiagnosing postoperative abscess, thus avoiding unnecessary intervention.^5,6^ Conversely, following just the imaging appearances can lead to unnecessary attempts to drain pseudo collections.^1^

Our case presents an unusual situation, where the presence of Surgicel^®^ masqueraded as abscess, while masking a different post-operative complication. The case highlights the importance of providing adequate operative details to the diagnostic radiologist. At the same time, it emphasizes the need for radiologists to be aware of criteria that may differentiate between abscess and haemostatic material—*e.g*. linear arrangement of tightly packed gas bubbles, lack of air–fluid level and lack of enhancing wall^7^ (Figure 1).

Another peculiarity of this case is the rapid disappearance of the gelatine sponge on the second CT study. Absorption of Surgicel^®^ depends upon several factors including the amount used, degree of saturation with blood, and the tissue bed.^2^ On imaging, it reportedly persists more than a month following surgery, both on CT and ultrasound^4,7^ ; yet in our case, there was complete resorption in the first postoperative week. This suggests that *in vivo* behaviour of Surgicel^®^ over time is still unpredictable and can vary widely.

## Learning points

1. The presence of Surgicel^®^ on postoperative CT studies can mimic abscesses, as previously shown, but it can also mask other postoperative complications.
2. Contrary to previous reports, the resorption time of Surgicel^®^ *in vivo* can be as short as 7 days.
3. Radiologists and interventionalists must be aware of the typical imaging appearances of haemostatic agents and of the unpredictability of the resorption time, to avoid performing unnecessary drainage procedures.
4. Clinicians requesting postoperative imaging must remember the paramount importance of providing precise clinical details, in particular regarding the intraoperative use of haemostatic agents.
